# Executive function in end-stage renal disease: Acute effects of hemodialysis and associations with clinical factors

**DOI:** 10.1371/journal.pone.0203424

**Published:** 2018-09-04

**Authors:** María del Mar Sánchez-Fernández, Gustavo A. Reyes del Paso, José Manuel Gil-Cunquero, María José Fernández-Serrano

**Affiliations:** 1 Department of Psychology, University of Jaén, Jaén, Spain; 2 Department of Nephrology, Jaén Hospital Complex, Jaén, Spain; University of Utah School of Medicine, UNITED STATES

## Abstract

**Objective:**

There is evidence of cognitive impairment in patients with end-stage renal disease in hemodialysis (ESRD-HD). However, few studies have exhaustively analyzed executive functions (EFs) in this population, especially considering the influence of a wide range of clinical variables. This study analyzes performance in different EF components in ESRD-HD patients compared to a group of healthy controls (HCs), in addition to the acute effects of HD and the associations of cognitive performance with clinical variables.

**Method:**

EFs were evaluated pre- and post-HD in 43 ESRD-HD patients and 42 HCs, using a battery of tests designed to assess EF domains. Age, schooling, mood and blood pressure were statistically controlled. Associations between performance and clinical factors were computed by correlations and hierarchical multiple regression analyses.

**Results:**

The performance of the ESRD-HD patients was significantly lower than that of HCs in all the EF domains except for planning. Group differences were marginally significant for reasoning. HD produced no acute changes in global performance, with improvements see only in inhibition and working memory. EF scores were positively associated with total number of months previously transplanted, body mass index (BMI), dry weight, and levels of hemoglobin, albumin, ferritin, calcium, phosphorus, sodium, urea, and creatinine.

**Conclusions:**

Global EF functioning was lower in ESRD-HD patients than in HCs. No major acute HD-related EF changes were detected. These findings underline the importance of an adequate nutritional status for maintaining executive functioning in ESRD-HD patients.

## Introduction

A range of disorders related to neuronal function have been observed in patients with end-stage renal disease (ESRD), including cognitive problems [1, 2]. Neuropsychological impairments including memory, learning, attention, and executive function (EF) deficits, have been described in ESRD patients on hemodialysis (ESRD-HD) [3, 4]. However, insufficient evidence is available on the extent and severity of such impairments [4].

EFs are involved in the generation, supervision, regulation, execution, and readjustment of behaviors needed to achieve complex objectives, especially those requiring a novel and creative approach [5, 6]. EFs can be subdivided into several domains: updating (e.g., fluency, working memory, and reasoning), inhibitory control, cognitive flexibility, planning, and decision-making [7]. Studies of EFs in ESRD-HD have focused on specific domains such as fluency, inhibitory control, or planning [8, 4]; information is lacking on other key domains, including verbal reasoning and decision-making. Methodological shortcomings of previous studies include a limited battery of tests with which to evaluate EF-related domains [4], small sample sizes and insufficient consideration of relevant sociodemographic variables (e.g., age and years of education) [9].

Research on possible acute changes in EFs produced by HD is available. Schneider et al. [4] reported post-dialysis improvements in cognitive flexibility and logical planning in comparison to pre-dialysis findings, but no improvement was observed in working memory, verbal fluency, or planning behavior. In addition, the possible influence of affective-emotional factors on EF performance [10, 11] has not been controlled for. However, the impact of negative emotional states (depression and anxiety) on the health-related quality of life in HD patients has been reported in previous studies [12]. Furthermore, inconsistent results have been reported in studies that addressed the relationship between cognitive and EF deficits in ESRD-HD and clinical (e.g., biochemical) variables, with no reports of correlations with hemoglobin, urea and creatinine [13] or counterintuitive associations between urea levels and neuropsychological scores [14]. These limitations suggest the need for further studies to provide a more exhaustive evaluation of EF deficits in ESRD, as well as possible associations between performance and clinical factors.

Using a large battery of tests to cover all EF components, the objectives of the study were: (a) to compare EFs between ESRD-HD patients and healthy controls (HCs), controlling for potentially influential sociodemographic and emotional variables; (b) to compare EFs before and after HD in ESRD-HD patients; and (c) to evaluate the associations between clinical variables and executive performance.

The study hypotheses were: (a) ESRD-HD patients would perform worse than HCs in all EF domains, (b) EFs scores would increase after HD in comparison to pre-HD levels, and (c) EFs scores would be associated with clinical and biochemical variables.

## Method

### Participants

The study included 43 patients who were receiving HD treatment (ESRD-HD) and 42 HCs. Seventeen patients were previously transplanted and seven were previously under peritoneal dialysis. All participants spoke Spanish as a first language. Inclusion criteria for the ESRD-HD group were: (1) receipt of HD for ≥ 3 months prior to the study; (2) receipt of three 4h HD sessions/week; (3) absence of any previous psychiatric diagnosis; (4) no history of traumatic brain injury or disorders affecting the CNS; and (5) absence of severe ophthalmic or auditory disease. HCs were recruited by using local advertising and a “snowball” approach, applying the same inclusion criteria as for the ESRD-HD group with the exception of criteria 1 and 2. Groups were matched for sex, age, and years of education (Table 1). Additionally, no differences in sex (*x*^2^ = 0.87, p = 0.35), age (t = -1.65; p = 0.11) or years of education (t = 0.75; p = 0.46) were observed between transplanted and non transplanted patients.

**Table 1:** Sociodemographic, biochemical, blood pressure and body composition data as a function of group.

Variables		ESRD-HD (n = 43)		HC (n = 42)	
		Mean	SD	Mean	SD
Age		51.72	9.310	51.83	6.525
Years of education		10.98	4.405	11.14	3.653
Sex *(%)*	Male	81.4		69.0	
	Female	18.6		31.0	
Hours pre-post ev.		47.67	36.47	35.31	6.61
Systolic blood pressure*		140.60	20.19	128.79	13.15
Diastolic blood pressure		85.85	11.76	83.76	11.78
Heart rate		70.34	11.06	69.51	8.40
BMI*		25.71	3.55	27.64	3.45
Dry weight kg*		70.45	14.27	80.04	13.52
Hemoglobin g/dL**		11.74	1.46	15.18	1.12
Hematocrit %**		35.17	4.55	45.26	3.14
Albumin g/dL**		3.96	0.27	4.37	0.32
Ferritin ng/mL**		399.49	156.45	123.56	57.98
Calcium mg/dL		9.44	1.26	9.57	0.27
Phosphorus mg/dL*		4.75	1.61	3.36	0.63
Sodium mEq/L		139.52	2.56	139.96	2.34
Urea mg/dL**		124.33	33.49	35.40	11.04
Creatinine mg/dL**		9.56	2.46	0.92	0.14

*p < .05

**p < .001

Note: Abbreviations and definitions: ESRD-HD: end-stage renal disease undergoing hemodialysis; HC: healthy control; Hours pre-post ev.: hours elapsed between pre- and post-evaluations; BMI: body mass index; Dry weight: ideal weight of individual with no excess fluid in blood.

### Executive function assessment

The tests included in this study are part of a broader neuropsychological protocol that includes evaluations of basic cognitive processes in ESRD-HD. The EF dimensions evaluated and instruments used were as follows:

*-Update*, i.e., the updating and monitoring of working memory contents. The tests for this domain were: (1) *Fluency*- Controlled Oral Word Association Test (COWAT) [15] to evaluate verbal fluency and Ruff Figural Fluency Test (RFFT) [16] to evaluate figural fluency. Dependent variables were the sum of words produced in two semantic categories, *semantic FAS* (animals and fruit), the sum of words produced from three letters, *phonological FAS* (F, A, S) for COWAT, and the *total number of original designs* produced for RFFT. (2) *Working memory*- two subtests of the Wechsler Adult Intelligence Scale (WAIS-III) [17]: Letters and numbers and Arithmetic; dependent variables were the number of *correct answers* in each test. (3) *Reasoning*- Similarities subtest of the WAIS-III [17]; the dependent measure was the number of correct answers.
*-Inhibition*, i.e., the inhibition of predominant or automatized responses that are inappropriate for current demands: the Five Digit Test (5DT) [18]. The dependent variable was the *inhibition score* (difference between execution time in condition 3 and the mean of conditions 1 and 2).
*-Cognitive flexibility*, i.e., the ability to alternate between different mental schemes, execution patterns, or tasks as a function of changing demands: the Wisconsin Card Sorting Test (WCST) [19] and the Five Digit Test (5DT) [18]; dependent variables were the *percentage of perseverative errors* in WCST, and the *shifting score* (difference between execution time in condition 4 and the mean of conditions 1 and 2) in 5DT.
*-Decision-making*, i.e., the ability to select the most advantageous option among a range of available alternatives: the Iowa Gambling Task (IGT) [20]; the dependent variable was *net IGT score*, obtained by subtracting the number of disadvantageous choices (blocks K’ and L’) from the number of advantageous choices (M’ and N’) in the 100 trials of the task.
*-Planning*: i.e., the ability to anticipate, rehearse, and execute complex behavioral sequences in a prospective manner: the Key Search subtest of the Behavioral Assessment of the Dysexecutive Syndrome (BADS) battery [21]; the dependent variable was the *profile score*, calculated by adding the raw scores of the test and applying the corrections listed in the administration manual.

Detailed information about these instruments can be obtained elsewhere [22, 7].

### Clinical assessment

Patients data were gathered from hospital records (Tables 1 and 2). The following clinical measures were included: (1) parameters of biochemistry frequently altered in ESRD-HD patients; (2) the types of renal replacement therapy received previously and the durations of treatment; and (3) the total Kt/v (dialysis dose) as an indicator the adequacy of dialysis. The glomerular filtration rate was not recorded because all participating patients were in stage 5 of the disease and had a GFR below 15 mL/min /1.73 m^2^. Body composition (% fat and body mass index-[BMI]) (Bodystat®1500 monitoring unit) and seven blood pressure readings (3M Tensocare B100 arm blood pressure monitor) were taken during the pre-HD evaluation.

**Table 2:** Clinical characteristics of the ESRD-HD group.

Variables	ESRD-HD (n = 43)	
	Mean	SD
Current months HD	42.00	61.53
Previous TX, *n (%)*	17 (39.53)	
Total months TX	42.53	81.04
Total months HD	58.88	70.25
Total months PD	4	10.46
Total months D	62.88	68.32
Total months RRT	105.42	114.56
Total Kt/v	1.69	0.41
Pre-HD hours	56.56	5.34
Post-HD hours	20.84	2.83
Diabetic patients, *n (%)*	3 (6.98)	
Hypertension patients, *n (%)*	38 (88.37)	
**Etiology of ESRD-HD, *n (%)***		
Glomerulonephritis	17 (39.5)	
Interstitial	2 (4.7)	
Hereditary	10 (23.3)	
Vascular	5 (11.6)	
Systemic	2 (4.7)	
Miscellaneous	1 (2.3)	
Diabetic	1 (2.3)	
Unknown	5 (11.6)	

Note: Abbreviations and definitions: ESRD-HD: end-stage renal disease undergoing hemodialysis; current months HD: number of months under current HD treatment; Previous TX, *n(%)*: total number (and percentage) of patients previously transplanted; Total months TX: total number of previous months in transplantation; Total months HD: total number of months under hemodialysis; Total months PD: total number of months under peritoneal dialysis; Total months D: total number of months under dialysis treatment (HD and/or PD); Total months RRT: total number of months under renal replacement therapy (hemodialysis, peritoneal dialysis, transplantation); Total Kt/v: calculated with the second-generation Daugirdas formula; Pre-HD hours: hours elapsed since most recent HD in the pre-HD evaluation; post-HD hours: hours since most recent HD in the post-HD evaluation.

Finally, because negative emotional states can affect cognitive performance, The Spanish version [23] of the *Hospital Anxiety and Depression Scale (HADS)* was used to evaluate anxiety and depression. Given the positive association between anxiety and depression severity (r = .61 in our study), and in order to reduce the number of variables in the regression analysis and keep alpha inflation at minimum, anxiety and depression scores were aggregated in to a global mood score.

### Procedure

**Fig 1:**
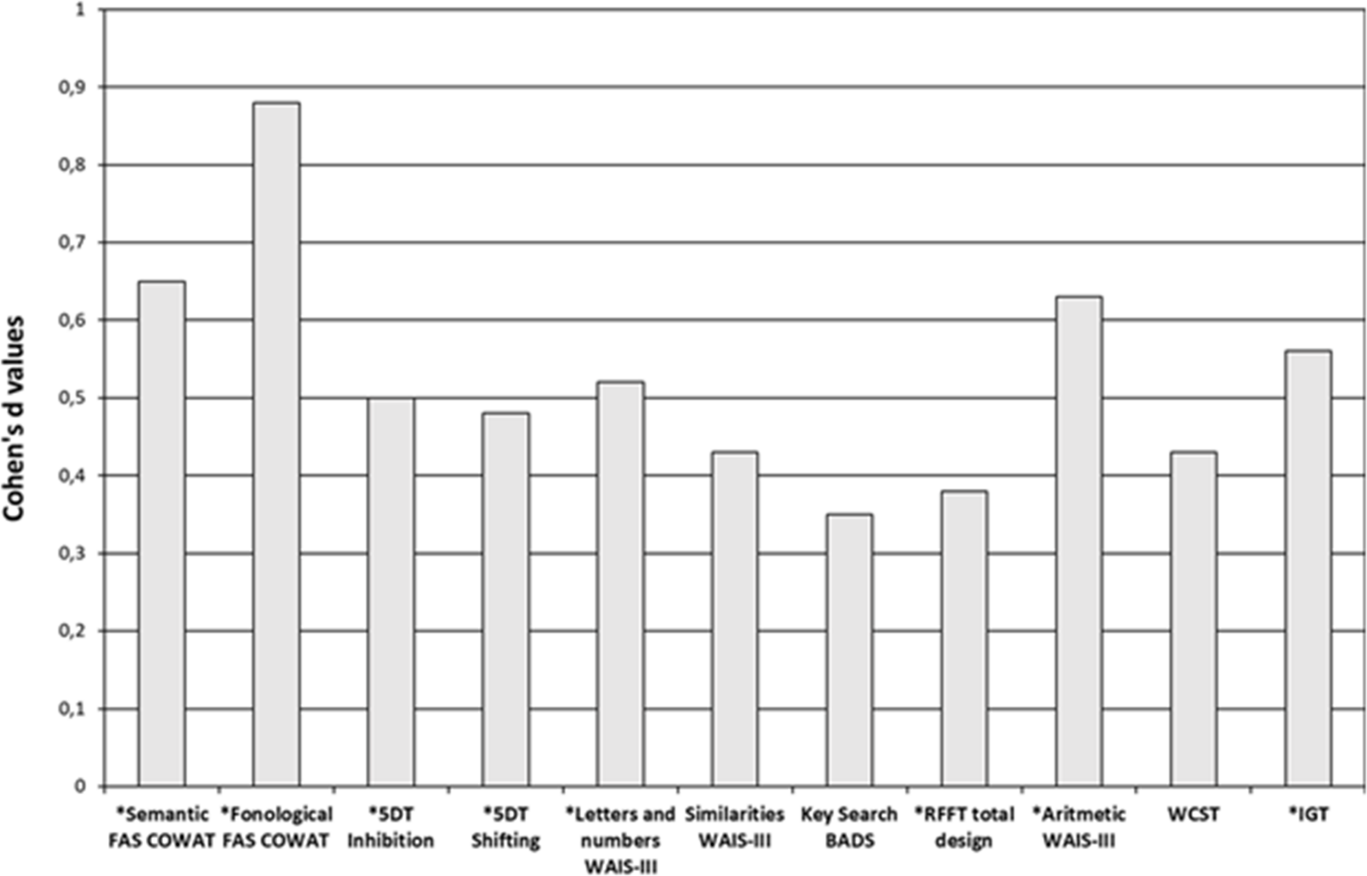
Cohen’s d values for group comparison of the dependent variables (the order of the tests in the figure follows their administration sequence).

Patients scheduled for HD were recruited from five hemodialysis centers in the provinces of Jaén and Granada (in Spain) and were individually evaluated in two sessions, before and after HD. All participants signed an informed consent form, which was approved by the Ethics Committee of the Jaén Hospital Complex. Evaluations were conducted in participants’ homes after ensuring adequate conditions for the assessment. Participants consumed no food, caffeine, alcohol, or tobacco during the tests. The pre-HD evaluation (before the HD session) was carried out in the hours before HD treatment, i.e., around 56 h after the last HD. The post-HD evaluation (after the HD session) was performed around 20 h after HD treatment, i.e., around 48 h after the pre-HD evaluation (Table 1). The two HC evaluations were separated by a similar period of time that in ESRD-HD patients. Tests were always administered in the same order, alternating between verbal and non-verbal tests and between more and less difficult tasks. Breaks (5 to 10 m) were taken when as necessary to minimize the potential for cognitive fatigue. Fig 1 shows the sequence in which the tests were administered.

### Statistical analyses

Multivariate analysis of variance (MANOVA) was used to analyze between-group differences during the first assessment (i.e., pre-HD). Age, years of education, mood and systolic blood pressure (to control for the higher levels in ESRD; see Table 1) were included as covariates. Differences between pre- and post-HD evaluations were analyzed by 2 × 2 repeated-measures ANOVA, with Group as the between-subject factor and the two evaluations as the repeated measure factor. A significant effect of the Group × Pre-Post interaction, associated with a greater post-HD increase in the ESRD-HD group, would indicate a positive effect of HD on performance. Adjusted square theta ($\eta_{p}^{2}$) and Cohen’s d were used as effect size indicators. Associations between performance and medical factors in the ESRD-HD group were computed in two steps. First, at an exploratory level, Pearson correlations were calculated; and second, hierarchical multiple regression analyses were performed using two blocks of data: (1) socio-demographic (age, years of education) and mood (simultaneously entered); and (2) clinical factors (stepwise method). We estimated the adjusted R^2^ of the predicted change in performance associated with each block.

## Results

### Group differences in executive performance

The MANOVA showed a main significant effect of Group (F(11, 69) = 2.79, p = 0.005, $\eta_{p}^{2}$ = 0.308). Effects of the covariates of years of education (F(11, 69) = 8.06, p<0.001, $\eta_{p}^{2}$ = 0.562) and age (F(11, 69) = 4.87, p<0.001, $\eta_{p}^{2}$ = 0.437) were also significant. Table 3 displays between-group values. Lower scores were obtained in the ESRD-HD group than in the HCs in two of the three fluency indexes (semantic FAS, phonological FAS), the two working memory indexes (Letters and Numbers and Arithmetic), the inhibitory control index (5DT inhibition), one of the two cognitive flexibility indexes (5DT shifting), and the decision-making index (IGT). Group differences were marginally significant for reasoning (Similarities). No group difference was observed in planning (Key Search). Size effects (Cohen´s d) ranged between 0.38 (figural fluency) and 0.88 (verbal fluency) (see Fig 1).

**Table 3:** Means (±SD), median, and 25^th^ and 75^th^ percentiles of neuropsychological variables for the two groups in the pre-HD evaluation.

Dominion	Dep. Variables	Group	Mean±SD	Median [25–75 percentiles]	F (1,79)	p	$\eta_{p}^{2}$
**Fluency**	**Semantic FAS COWAT**	**ESRD-HD**	30.19±7.65	28 [25–35]	10.51	0.002	0.117
		**HC**	35.12±7.64	34 [30–40.25]			
	**Phonological FAS** **COWAT**	**ESRD-HD**	27.74±12.80	25 [19–34]	22.01	<0.001	0.218
		**HC**	37.93±10.26	40 [28–46.25]			
	**RFFT total design**	**ESRD-HD**	68.26±19.66	68 [50–85]	2.76	0.100	0.034
		**HC**	75.69±19.25	75.50 [67.25–90.25]			
**Working memory**	**Letters and Numbers** **WAIS-III**	**ESRD-HD**	7.91±3.02	8 [6–10]	6.46	0.013	0.076
		**HC**	9.45±2.84	9 [7.75–11]			
	**Arithmetic** **WAIS-III**	**ESRD-HD**	10.70±3.16	10 [8–13]	10.18	0.002	0.114
		**HC**	12.83±3.61	13 [10–15.25]			
**Reasoning**	**Similarities** **WAIS-III**	**ESRD-HD**	15.21±5.29	16 [13–19]	3.89	0.052	0.047
		**HC**	17.48±5.22	17 [13.75–22.25]			
**Inhibition**	**5DT Inhibition**	**ESRD-HD**	18.17±7.32	17 [13–23]	7.33	0.008	0.085
		**HC**	14.89±5.68	15 [11.37–18.12]			
**Cognitive flexibility**	**5DT Shifting**	**ESRD-HD**	38.73±16.61	36 [25–52.50]	6.87	0.011	0.080
		**HC**	31.30±14.16	29 [22.50–36.25]			
	**WCST**	**ESRD-HD**	20.95±13.71	19 [11–27]	3.64	0.06	0.044
		**HC**	15.86±9.31	13 [9–22]			
**Decision-making**	**IGT**	**ESRD-HD**	-1.12±7.49	-2 [–6–4]	4.85	0.030	0.058
		**HC**	5.79±15.67	4.50 [-6.50–14]			
**Planning**	**Key search** **BADS**	**ESRD-HD**	1.63±1.79	1 [0–4]	1.95	0.166	0.024
		**HC**	2.21±1.47	2 [1–4]			

Note: Results of group comparisons after controlling for age, years of education, mood and blood pressure are also included. ESRD-HD: end-stage renal disease undergoing hemodialysis; HC: healthy control.

### Effect of dialysis

In these analyses, the Group factor (global execution during both evaluations) was significant for fluency (semantic FAS: p = 0.01, $\eta_{p}^{2}$ = 0.084; phonological FAS: p<0.001, $\eta_{p}^{2}$ = 0.15), cognitive flexibility (5DT: p = 0.04, $\eta_{p}^{2}$ = 0.052), and working memory (Letters and numbers: p = 0.01, $\eta_{p}^{2}$ = 0.09, Arithmetic: p = 0.03, $\eta_{p}^{2}$ = 0.053). Group differences were marginally significant for reasoning (Similarities: p = 0.06, $\eta_{p}^{2}$ = 0.042), fluency (RFFT total design: p = 0.07, $\eta_{p}^{2}$ = 0.04), and cognitive flexibility (WCST: p = 0.06, $\eta_{p}^{2}$ = 0.042). In all cases, the execution was worse in the ESRD-HD *versus* HC group.

Performance improved between the pre- and post-HD assessments in both groups. Specifically, performance increases were seen in fluency, working memory, reasoning, inhibitory control, cognitive flexibility, and planning indexes in both groups (Table 4). The Group × Pre-Post-HD interaction was significant for the inhibition index 5DT (F(1,83) = 6.59, p = 0.01, $\eta_{p}^{2}$ = 0.074) and the working memory index of Arithmetic (F(1,83) = 5.43, p = 0.02, $\eta_{p}^{2}$ = 0.061). Analysis of these interactions showed that patients improved their inhibition performance in 5DT (F(1,42) = 25.32, p<0.001, $\eta_{p}^{2}$ = 0.376), and working memory performance in Arithmetic (F(1,42) = 16.24, p<0.001, $\eta_{p}^{2}$ = 0.279); meanwhile, HCs showed no significant change in either 5DT inhibition (F(1,41) = 2.17, p = 0.148, $\eta_{p}^{2}$ = 0.050) or Arithmetic (F(1,41) = 3.73, p = 0.060, $\eta_{p}^{2}$ = 0.083).

**Table 4:** Means (±SD) of the pre- and post- cognitive evaluations and statistics for the main repeated measures factor.

Domain	Dep. Variables	Group	Mean±SD Pre-	Mean±SD Post	F (1,83)	p	Ƞ^2^_p_
**Fluency**	**Semantic FAS** **COWAT**	**ESRD-HD**	30.19± 7.65	33.51±9.17	24.48	<0.001	0.228
		**HC**	35.12± 7.64	37.31 ±6.21			
	**Phonologic FAS** **COWAT**	**ESRD-HD**	27.74±12.80	31.98±12.46	28.99	<0.001	0.259
		**HC**	37.93±10.26	40.29±10.39			
	**RFFT total design**	**ESRD-HD**	68.26±19.66	80.65±25.60	114.48	<0.001	0.580
		**HC**	75.69±19.25	89.83±22.75			
**Working memory**	**Letters and Numbers** **WAIS-III**	**ESRD-HD**	7.91± 3.02	8.30± 2.83	8.81	0.01	0.096
		**HC**	9.45± 2.84	10.26±3.17			
	**Arithmetic** **WAIS-III**	**ESRD-HD**	10.70±3.16	12.16±3.66	19.47	<0.001	0.190
		**HC**	12.83±3.615	13.29±4.06			
**Reasoning**	**Similarities** **WAIS-III**	**ESRD-HD**	15.21±5.29	15.98±4.82	4.01	0.04	0.046
		**HC**	17.48±5.22	17.81±5.19			
**Inhibition**	**5DT interference**	**ESRD-HD**	18.17±7.32	14.14±6.36	21.41	<0.001	0.205
		**HC**	14.89±5.68	13.74±4.91			
**Cognitive flexibility**	**5DT** **shifting**	**ESRD-HD**	38.73±16.61	27.19±14.46	91.51	<0.001	0.524
		**HC**	31.30±14.16	22.50±10.01			
	**WCST**	**ESRD-HD**	20.95±13.71	15.83±12.01	23.08	<0.001	0.220
		**HC**	15.86±9.31	11.79±8.55			
**Planning**	**Key search** **BADS**	**ESRD-HD**	1.63±1.79	1.81±1.68	4.81	0.03	0.055
		**HC**	2.21±1.47	2.36±1.48			

Note: ESRD-HD: End-stage renal disease undergoing hemodialysis; HC: healthy control. Only variables that showed significant changes were displayed.

### Associations between neuropsychological performance and clinical parameters in ESRD-HD

#### Exploratory correlation analyses

*Total number of months previously transplanted* was positively associated with decision-making (IGT: r = 0.36, p = 0.02); *BMI* was positively associated with working memory (Arithmetic: r = 0.34, p = 0.02); *dry weight* was positively associated with working memory (Arithmetic: r = 0.44, p = 0.01); *hemoglobin* was positively associated with fluency (RFFT total design: r = 0.22, p = 0.042); *albumin* was positively associated with working memory (Letters and Numbers: r = 0.32, p = 0.035); *ferritin* was positively associated with lower inhibition (5DT: r = 0.32, p = 0.03); *calcium* was positively associated with working memory (Letters and Numbers: r = 0.32, p = 0.03); *phosphorus* was positively associated with working memory (Arithmetic: r = 0.35, p = 0.02); *sodium* was positively associated with working memory (Arithmetic: r = 0.43, p = 0.01) and decision-making (IGT: r = 0.31, p = 0.05); *urea* was positively associated with working memory (Arithmetic: r = 0.38, p = 0.01), and reasoning (Similarities: r = 0.42, p = 0.01); and *creatinine* was positively associated with working memory (Arithmetic: r = 0.31, p = 0.04).

#### Multiple regression analyses to predict executive functioning

The following significant regression models were obtained after controlling for the effects of socio-demographic variables and mood: *Sodium* was positively associated with (1) semantic FAS (*β* = 0.26, r^2^ = 0.06, t = 2.04, p = 0.05) and (2) Arithmetic (*β =* 0.48, r^2^ = 0.22, t = 4.19, p<0.001). In the latter case, dry weight was included in a second model with and showed positive associations (*β =* 0.48, t = 4.57, p<0.001 for sodium and *β* = 0.31, t = 2.88, p = 0.01 for dry weight; r^2^ = 0.09). *Calcium* was positively associated with Letters and numbers (*β =* 0.37, r^2^ = 0.14, t = 3.37, p = 0.01); BMI was included in a second model and showed negative associations (*β =* 0.34, t = 3.23, p = 0.01 for calcium and *β =* -0.25, t = -2.25, p = 0.03 for BMI; r^2^ = 0.05), and hematocrit was included in a third model and showed positive associations (*β* = 0.29, t = 2.87, p = 0.01 for calcium, *β =* -0.30, t = -2.82, p = 0.01 for BMI, and *β =* 0.24, t = 2.28, p = 0.03 for hematocrit; r^2^ = 0.05). *Urea* was positively associated with Similarities (*β =* 0.30, r^2^ = 0.09, t = 2.41, p = 0.02) and *ferritin* was positively associated with lower inhibition in 5DT (*β* = 0.27, r^2^ = 0.07, t = 2.13, p = 0.04).

## Discussion

This study explored EFs in ESRD-HD patients by using a comprehensive test battery covering the domains of updating (fluency, working memory, and reasoning), inhibitory control, cognitive flexibility, planning and decision-making. Patients performed worse than HCs in terms of fluency, working memory, inhibitory control, cognitive flexibility, and decision-making. These results are in line with previous reports. Regarding verbal fluency, Harciarek et al. [24] found a reduced rate of word production of in phonological fluency tasks, while Post et al. [25], also using the COWAT, reported worse performance in phonological and semantic verbal fluency. Regarding working memory, Anwar et al. [8] and Costa et al. [13] observed lower performance by ESRD-HD patients relative to HCs. Previous studies [3, 25] also found impaired inhibitory control in ESRD-HD patients. With respect to cognitive flexibility, Anwar et al. [8] and Post et al. [25] noted impairment in ESRD-HD patients on the Trail Making Test B. Concerning reasoning, Pereira et al. [26] reported significant impairments in non-verbal fluency reasoning by ESRD-HD patients, but they did not include a control group (results were compared with normative data); in addition, their assessments were conducted during HD sessions, and thus may have been affected by the associated distractions/interferences. It should be taken into account that while non-verbal fluency reasoning was evaluated in their study, we investigated verbal fluency reasoning. This report is the first of reduced decision-making performance (IGT) in ESRD-HD patients, but we found no differences with HCs in terms of planning strategies, as measured by the BADS test, thus confirming the findings of Schneider, Malecki, et al. [4].

The second objective of our study was to establish the acute effects of HD. Both of our groups showed improvement in several EF indexes between the first and second assessments, attributable to learning processes (experience and familiarization gained in the first round of the tests). Regarding group differences in pre-post evaluations, ESRD-HD patients showed a greater post-HD improvement in inhibition (5DT) and working memory (Arithmetic) in comparison to HCs. The study hypothesis was therefore not completely fulfilled, as no differential global improvement was observed after HD in the ESRD group. These results are in partial agreement with the findings of Schneider et al. [4], who reported no changes in post-HD performance in verbal fluency, working memory, or planning behavior, but described improvements in cognitive change and logical activity planning.

The third objective was to analyze associations between EF performance and clinical and biochemical variables [27]. Decision-making performance (IGT) was positively associated with the total number of months previously transplanted. More improved cognitive performance in transplanted patients than in ESRD-HD patients has been reported in memory indexes [28], psychomotor speed, visual planning, learned material recall, abstract thinking [29], processing speed, attention, short-term memory, convergent thinking, and EFs [30]. In a prospective study of ESRD-HD patients undergoing transplantation, Gupta et al. [31] found significant post-transplant improvements in memory and EF scores at 3 months, simultaneous with improvements in the integrity of brain white matter in areas associated with memory and EFs. These data suggest that cognition and brain structural abnormalities observed in ESRD-HD patients are at least partially reversible and are to some degree dependent on the renal replacement therapy modality administered.

With respect to body composition parameters, BMI and dry weight showed a positive association with working memory (Arithmetic). Radić et al. [32] observed superior cognitive performance in working memory, visual orientation, and convergent thinking in individuals with BMI ≥ 23, while lower intradialysis weight was found to predict poorer performance in attention tests [13]. Giang et al. [33] proposed that poorer cognitive function in underweight ESRD-HD patients may in part be explained by their high incidence of malnutrition and, therefore, greater susceptibility to dialysis-related toxicity. Hence, a higher BMI may be associated with superior EF functioning in these patients.

With regard to the relationships between biochemical variables and EF indexes, we highlight the association of nutritional status and inflammatory markers with working memory, inhibition, reasoning, and decision-making. Albumin was positively associated with working memory (Letters and numbers), confirming previous findings [32] on the relationship between nutritional status and cognitive performance in ESRD-HD. Albumin, with normal values ranging between 4 and 4.5 g/dL (mean of our ESRD-HD group = 3.96 g/dL) is a nutritional marker and predictor of morbidity and mortality in this population [34]. Higher albumin levels indicate better nutrition and may therefore predict superior neuropsychological status. Calcium and phosphorus levels were also positively associated with working memory indexes (Letters and number for calcium, Arithmetic for phosphorus). Higher calcium levels have previously been related to improved perception, visual memory, and visual-constructive abilities [14]. Ferritin was positively associated with the inhibition index (5DT, where higher score means worse execution). ESRD-HD is associated with a chronic inflammatory status [35] characterized by iron deficiency, with normal-high serum ferritin levels [36]. Normal ferritin levels range between 30 and 300, whereas the mean level was 399 in the present patients. This elevated value may indicate the presence of inflammation, which can impair performance in the inhibition test. Sodium was positively associated with decision-making (IGT) and working memory (Arithmetic). An association between hyponatremia and cognitive impairment has previously been reported in ERT-HD patients [13, 37] and those with cardiovascular disease [38]. Reduced sodium levels were found to predict poor results in inhibition tests [13], corroborating the effects of hyponatremia on EFs.

Urea was positively associated with working memory indexes (Arithmetic) and reasoning (Similarities) in this study. An association between increased urea and EF improvement was also reported by Griva et al. [14] in ESRD patients. In an investigation of the effects of HD frequency on cognitive performance, Kurella Tamura et al. [39] found that higher HD frequency did not improve EFs, suggesting that residual uremia is not the main factor responsible for neuropsychological impairment in these patients. However, the negative effect of uremic status on cognitive performance is well documented [1, 40, 41]. In conclusion, the contribution of urea to executive functioning in ESRD-HD patients remains unclear, and is likely mediated by other biochemical-clinical variables and nutritional status.

Other biochemical markers commonly related to neuropsychological performance are hematocrit and hemoglobin. In this investigation, hematocrit was associated with working memory (Letters and numbers), in line with previous findings [42]. Hemoglobin, which has previously been related to neuropsychological performance in ESRD-HD [3, 8], was associated with figural fluency (RFFT total design) in our study. No association was observed between Kt/v value and performance. Likewise, Giang et al. [33] found no evidence of an association between low Kt/v levels and poorer memory or EF execution in ESRD-HD patients.

A major strength of the present study was the exhaustive evaluation of EF components, which have generally been analyzed in only a partial manner in previous investigations. Several authors have focused on the evaluation of executive indexes such as working memory, verbal fluency, cognitive flexibility, or inhibitory control [3, 8, 13], but there has been little research on behavioral planning [4] or decision-making, among other domains. Adequate decision-making by ESRD patients may be crucial in the choice of renal replacement therapy, and in terms of feeding, taking medication, performing daily life activities, or attending dialysis sessions, all of which significantly influence quality of life and survival. We also analyzed the effect of HD treatment on cognitive performance, and the possible influence of variables related to ESRD and HD. The few previous investigations that compared performance before and after HD had certain limitations, including a lower educational level of patients than of HCs [4]. Finally, the effects of age, schooling, mood and blood pressure on performance were controlled for in the present study.

In summary, the results of this study reveal a lower global EF performance by ESRD-HD patients than by matched HCs participants in terms of fluency, working memory, inhibitory control, cognitive flexibility, and decision-making. No differences were observed in planning. Comparisons of performance before and after HD showed no global improvement, with only partial gains for inhibition (5DT) and working memory (Arithmetic). With respect to the influence of clinical factors, we highlight the improved performance by patients with a history of transplantation. Many of the associations detected underscore the importance of good nutritional status for achieving good executive functioning.
